# Neonatal Alcohol Exposure in Mice Induces Select Differentiation- and Apoptosis-Related Chromatin Changes Both Independent of and Dependent on Sex

**DOI:** 10.3389/fgene.2020.00035

**Published:** 2020-02-11

**Authors:** Samantha L. Schaffner, Alexandre A. Lussier, Jessica A. Baker, Dan Goldowitz, Kristin M. Hamre, Michael S. Kobor

**Affiliations:** ^1^Centre for Molecular Medicine and Therapeutics, British Columbia Children’s Hospital Research Institute – Department of Medical Genetics, University of British Columbia, Vancouver, BC, Canada; ^2^Department of Anatomy and Neurobiology, University of Tennessee Health Science Center, Memphis, TN, United States; ^3^Human Early Learning Partnership, School of Population and Public Health, University of British Columbia, Vancouver, BC, Canada

**Keywords:** neonatal alcohol exposure, epigenetics, chromatin, apoptosis, sex differences

## Abstract

Prenatal alcohol exposure (PAE) affects many aspects of physiology and behavior, including brain development. Specifically, ethanol can influence expression of genes important for brain growth, including chromatin modifiers. Ethanol can also increase apoptotic cell death in the brain and alter epigenetic profiles such as modifications to histones and DNA methylation. Although differential sex outcomes and disruptions to the function of multiple brain regions have been reported in fetal alcohol spectrum disorder (FASD), the majority of our knowledge on molecular epigenetic and apoptotic dysregulation in PAE is based on data from males and is sometimes limited to assessments of the whole brain or one brain region. Here, we examined histone modifications, DNA methylation, and expression of genes involved in differentiation and proliferation related-chromatin modifications and apoptosis in the cerebral cortex and cerebellum of C57BL/6J mice exposed to an acute alcohol challenge on postnatal day 7, with a focus on differential outcomes between sexes and brain regions. We found that neonatal alcohol exposure altered histone modifications, and impacted expression of a select few chromatin modifier and apoptotic genes in both the cortex and cerebellum. The results were observed primarily in a sex-independent manner, although some additional trends toward sexual dimorphisms were observed. Alcohol exposure induced trends toward increased bulk H3K4me3 levels, increased *Kmt2e* expression, and elevated levels of *Casp6* mRNA and bulk γH2A.X. Additional trends indicated that ethanol may impact *Kdm4a* promoter DNA methylation levels and bulk levels of the histone variant H2A.Z, although further studies are needed. We comprehensively examined effects of ethanol exposure across different sexes and brain regions, and our results suggest that major impacts of ethanol on bulk chromatin modifications underlying differentiation and apoptosis may be broadly applicable across the rodent cortex and cerebellum in both sexes.

## Introduction

Prenatal alcohol exposure (PAE) can lead to lifelong physical, behavioral, and cognitive deficits (Stratton et al., 1996; Hoyme et al., 2016). The lasting condition resulting from PAE includes a broad range of symptoms and is termed fetal alcohol spectrum disorder (FASD), affecting an estimated 0.8% of children worldwide, 1%–5% of children in the USA, and 0.5%–1.8% of children in Canada (Lange et al., 2017; Popova et al., 2017; May et al., 2018; Popova et al., 2019).

During mammalian brain development, periods of neurogenesis, gliogenesis, differentiation, and normal apoptotic cell death occur to establish and maintain a healthy population of neurons and glia (Stiles and Jernigan, 2010). Chromatin and epigenetic factors such as DNA methylation, histone posttranslational modifications (PTMs), and histone variant exchange help to orchestrate and maintain these processes and guide expression of the genes controlling them. For example, DNA methylation and hydroxymethylation patterns at CpG and CpH sites can affect the potential of neural progenitors and lock in neural identity (Kriaucionis and Heintz, 2009; Guo et al., 2014; Stricker and Gotz, 2018). Histone PTMs such as acetylation, methylation, phosphorylation, ubiquitination, and SUMOylation also affect chromatin state and accessibility of genes for transcription (Kouzarides, 2007; Bannister and Kouzarides, 2011; Bowman and Poirier, 2015). Histone acetylation and methylation of histone H3 at lysines 4 (H3K4), 9 (H3K9), and 27 (H3K27) are of particular interest due to their role in regulating developmental transcription, differentiation, and pluripotency, including neuronal differentiation and the neurogenesis/gliogenesis switch (Vastenhouw and Schier, 2012; Gavin et al., 2017). Additionally, core histones H2A and H3 can be exchanged for different variants that affect chromatin structure and can impact brain development (Jin et al., 2005; Henikoff and Smith, 2015). Exchange of histone H2A for the variant H2A.Z, for instance, influences transcription of many mammalian genes and is crucial for overall vertebrate development, with specific roles identified in brain development and synaptic plasticity (Faast et al., 2001; Ridgway et al., 2004; Zovkic et al., 2014; Shen et al., 2018). These epigenetic cues may be most vulnerable to disruptions during the third trimester of human development and up to approximately the first year of life, which represents a critical period of rapid brain growth (Dobbing and Sands, 1974). In mice, this period of rapid development occurs after birth and extends until postnatal day 21 (Butts et al., 2014).

Previous work has demonstrated that alcohol exposure, among other things, affects the landscape of epigenetic marks, expression of epigenetic modifiers, and rate of apoptosis in cellular and animal models. In neural stem cells, ethanol reduces H3K4me3 and H3K27me3 levels at the promoters of neuronal specification genes, which may disrupt neurogenesis (Veazey et al., 2013). In male mice, increased expression of lysine methyltransferase enzymes is increased after alcohol exposure (Hashimoto et al., 2017). Additionally, neonatal alcohol exposure can accelerate neuronal apoptosis rates and increase γH2A.X levels (Subbanna et al., 2013; Goldowitz et al., 2014; Subbanna et al., 2014).

While these studies support the idea that chromatin dysregulation may be involved in the pathogenesis of PAE, most rodent work has not discriminated between specific brain regions (Guo et al., 2011; Subbanna et al., 2013; Goldowitz et al., 2014; Nagre et al., 2015). As epigenetic modifications vary greatly between tissues and cell types, brain region remains an important consideration for future work in neurodevelopmental epigenetics (Ohgane et al., 2008). PAE affects almost every region of the brain. Among other defects, ethanol exposure can result in an altered or absent corpus callosum, and reductions in hippocampal, cortical, and cerebellar volume (Lebel et al., 2011). Additionally, ethanol induces changes to the function of the hypothalamic-pituitary-adrenal (HPA) axis, such as increased activation of hypothalamic neurons (Weinberg et al., 2008). The cortex and cerebellum represent two areas directly related to neuropsychological phenotypes of FASD, including deficits in cognition, learning, attention, and language (Kandel et al., 2000; Wolf et al., 2009). These brain regions may also be particularly vulnerable to effects of alcohol on postnatal day 7 in mice, as neurogenesis and specialized cell differentiation occur in both of these brain regions during the human third trimester (Malik et al., 2013; Butts et al., 2014).

In addition to examining mainly one brain region at a time, existing research on PAE epigenetics has heavily focused on males, though sex differences exist during typical brain development and have also been reported in later life phenotypic outcomes of FASD in humans (Handa et al., 1985; Minetti and Fulginiti, 1991; Wilson et al., 1996; Hellemans et al., 2010a; Hellemans et al., 2010b; Ratnu et al., 2017). Some additional rodent studies have investigated the effects of PAE on females; however, only a few have assessed both males and females concurrently (Lussier et al., 2015; Amiri et al., 2019).

In this study, we conducted a targeted analysis of chromatin modifiers and apoptotic genes that may be involved in mediating the early effects of ethanol-induced neurodegeneration in the cortex and cerebellum, with an emphasis on sex- and region-specific effects. We investigated DNA methylation, histone PTMs, and the expression of chromatin modifying enzymes and of histone variant H2A.Z. Our analysis revealed that ethanol exposure was associated with elevated levels of H3K4me3 and expression of the enzyme that catalyzes addition of this mark, *Kmt2e*. Additionally, we observed trends toward increased, bulk H2A.Z levels, *Kdm4a* promoter DNA methylation, bulk γH2A.X levels and *Casp6* transcription upon ethanol exposure, and sexually dimorphic patterns of H2B-S14P in ethanol-exposed animals. The strongest effects of ethanol on H3K4me3, *Kmt2e*, *Casp6*, and γH2A.X trended consistently across sexes and brain regions, suggesting that major effects of neonatal binge ethanol exposure on chromatin may be applicable across sexes and brain regions.

## Materials and Methods

### Animals and Ethanol Exposure

The animal model used in this study represents an acute neonatal binge exposure, as only two high doses of ethanol were administered. These resulted in blood alcohol levels around 350 mg/dl, as previously reported (Goldowitz et al., 2014). This level is much higher than would be observed in typical human binge drinking but is appropriate for a rodent acute exposure paradigm due to the rapid alcohol metabolism of mice (Heatley and Crane, 1990; Cederbaum, 2012). This high dose and the harvesting of brain tissues 7 h after ethanol exposure allowed us to study the acute effects of ethanol on the developing brain, such as cell death (Kleiber et al., 2014).

All mouse work was conducted at the University of Tennessee Health Science Center (UTHSC) and experimental protocols were performed with the approval of the Institutional Animal Care and Use Committee at UTHSC and in accordance with the Guide for the Care and Use of Laboratory Animals. Adult C57BL/6J mice (over 90 days of age) were maintained on a 12:12 hour light-dark cycle and given food and water ad libitum. Males and females were mated, and vaginal plugs were used to detect pregnancy. The mice were observed several times per day and the day of birth was classified as postnatal day 0. Animals were exposed to ethanol on postnatal day 7 (P7) as previously published (Olney et al., 2002; Goldowitz et al., 2014). In brief, mice were given a total exposure of 5.0 g/kg of ethanol (20% v/v in sterile saline) given in two equal doses of 2.5 g/kg separated by 2 h. Controls were given isovolumetric saline and all exposure was given *via* subcutaneous injection. The first injection was given between 9 a.m. and 10 a.m., and the second injection between 11 a.m. and 12 p.m. A total of five litters were collected, with five to six mice taken from each (**Table S1**); males and females from each litter were randomly assigned to either the ethanol-exposed or control group.

### Tissue Collection

Seven hours after the first injection, mice were sacrificed by cervical dislocation. The brains were dissected and separated into cortex and cerebellum. Each region was placed in a microfuge tube, flash frozen in liquid nitrogen and stored at −80˚C until shipped to the University of British Columbia for analyses.

### Tissue Processing and DNA/RNA/Histone Extractions

Frozen brain specimens were thawed on dry ice and dissected to ~2 mm pieces. Tissues were homogenized using 20 G and 23 G needles in Qiagen^â^ Buffer RLT Plus (QIAGEN Inc., Germantown, Maryland, USA) with β-mercaptoethanol. DNA and RNA extractions were then performed with the Qiagen^®^ AllPrep DNA/RNA Mini Kit as per manufacturer's instructions. Nuclear histones were extracted from the cerebral cortex and cerebellum of P7 mice using previously described methods (Rumbaugh and Miller, 2011).

### Protein Blot Analysis of Histone Marks

Histones were loaded onto 15% SDS-polyacrylamide gels and separated by electrophoresis. Proteins were transferred onto nitrocellulose membranes and blocked with 5% milk in Tween-tris buffered saline (TTBS) for 2 h at room temperature. Primary antibodies were used to measure modified histone and core histone levels (**Table S2**). Membranes were incubated with rabbit primary antibody for 2 h at room temperature, followed by a 16 h incubation at 4°C with mouse primary antibody. They were then incubated with secondary antibodies against mouse (1/10,000) and rabbit (1/15,000) for 1 h at room temperature (**Table S2**). Membranes were washed for 3× 5 min between incubations with 0.1% Tween-20 Tris-Buffered Saline. Bands were imaged using the Li-Cor Odyssey scanner and histone modification/histone variant levels were quantified using the ImageStudio Lite software (Li-Cor Biosciences, Lincoln, Nebraska, USA). Histone modification levels are reported as modification/total histone (e.g. H3K27me3/total H3). H2A.Z levels are reported as H2A.Z/H4.

### Gene Expression Profiling

Gene expression was initially profiled using the Mouse “Apoptosis” and “Epigenetic Chromatin Modification Enzyme” RT² Profiler™ PCR Arrays, which interrogate 84 key genes involved in these processes (PAMM-012ZR, PAMM-085ZR; SABiosciences, QIAGEN Inc., Germantown, Maryland, USA). The apoptosis array was performed as per manufacturer's instructions on RNA from the cortex (three ethanol; three control) and cerebellum (three ethanol; two control) of male mice, while the chromatin enzyme array was performed on the cortex of male mice (three ethanol; three control). Reactions were run on a Qiagen^®^ Rotor-Gene Q real-time PCR cycler, and cycle threshold analysis was performed with SABiosciences software provided with the arrays.

Reverse transcription quantitative polymerase chain reaction (RT-qPCR) analysis was performed to validate major changes in gene expression observed in the PCR arrays using the full set of samples, and to interrogate histone variant expression. Two apoptotic genes, four chromatin modifier genes from the arrays, and one histone variant gene were analyzed. 1 µg RNA per sample was converted to cDNA using the Qiagen^®^ QuantiTect^®^ Reverse Transcription Kit as per manufacturer's instructions. A dilution standard curve was created for each primer set to determine optimal cDNA template concentration and background threshold. 6-µl diluted template DNA per reaction was added to 9-µl master mix containing PerfeCTa SYBR^®^ Green FastMix (Quantabio, Beverly, Massachusetts, USA), forward primer, and reverse primer (**Table S3**). Reactions were run in triplicate on a Qiagen^®^ RotorGene Q real-time PCR cycler, and subsequent cycle threshold analysis was performed with the accompanying SABiosciences software (**Figure S4**). Expression fold change values were calculated using the 2^(ΔΔCt)^ method, normalized to levels of phosphoglycerate kinase 1 (*Pgk1*; Winer et al., 1999).

### Sodium Bisulfite Conversion and Pyrosequencing

500 ng genomic DNA per sample was sodium bisulfite converted with the EZ DNA Methylation™ Kit (Zymo Research Corp., Irvine, California, USA), and subsequently analyzed by spectrophotometry for concentration and quality. Converted DNA was diluted 10-fold and 1 µl was used per 30 µl PCR reaction. Reactions were amplified with HotStarTaq DNA Polymerase (QIAGEN Inc., Germantown, Maryland, USA) for 45 cycles at an annealing temperature of 58°C (see **Table S3** for primer information). PCR samples were visualized on a 1% agarose gel postamplification to ensure integrity.

Pyrosequencing assays were designed with PyroMark^®^ Assay Design 2.0 software (QIAGEN Inc., Germantown, Maryland, USA) for genes from expression analysis which passed quality standards including regional CpG density, PCR primer specificity, and amplicon length: *Ltbr*, *Kmt2e*, and *Kdm4a* (**Tables S3** and **S4**). 15 µl PCR reaction, 65 µl binding buffer, and 12 µl sequencing reaction mix per sample was prepared with PyroMark^®^ Gold Q96 Reagents according to the manufacturer's instructions. Sequencing was performed with the PyroMark^®^ Q96 ID (QIAGEN Inc., Germantown, Maryland, USA).

### Statistical Analyses

Welch's *t*-tests were used to compare gene expression (normalized fold change values) between control and ethanol-treated mice, and DNA methylation at each CpG site. The effects of sex and brain region on overall gene expression changes were assessed by ANOVA (expression fold change ~ treatment + sex + brain region) and Tukey's Honestly Significant Difference (HSD) test. All *p-*values < 0.05 were considered statistically significant.

## Results

### Ethanol Induced Differential Expression of a Limited Set of Chromatin Modifiers in the Neonatal Mouse Brain

We hypothesized that acute alcohol exposure at postnatal day 7 could affect mRNA expression levels of chromatin-modifying enzymes, due to the importance of establishing epigenetic marks associated with neurogenesis and gliogenesis. PAE has been shown to alter histone modifications and DNA methylation, supporting this hypothesis (reviewed in Basavarajappa and Subbanna, 2016; Gavin et al., 2017; Lussier et al., 2017). Our experiments focused on the cortex and cerebellum because these brain regions control processes vulnerable to ethanol-induced dysfunction.

We assayed mRNA levels of 84 genes encoding chromatin-modifying enzymes using the SABiosciences qPCR array in the cortex of male ethanol and control animals (**Table S6**, n = 3 per group). We identified alterations in the expression of four genes that passed a liberal threshold of *p* < 0.1, selected to identify trends in the face of low sample numbers: lysine methyltransferase 2E (*Kmt2e*; *p* = 0.04), lysine methyltransferase 5A (*Kmt5a*; *p* = 0.08), lysine demethylase 4A (*Kdm4a*; *p* = 0.03), and DNA methyltransferase 3B (*Dnmt3b*; *p* = 0.06). In addition to the chromatin modifiers assessed by qPCR array, we also investigated the impact of ethanol on expression of the *H2afz* gene, encoding histone variant H2A.Z, due to previous work that noted altered *H2afz* mRNA and H2A.Z protein levels in response to alcohol (Gretzinger et al., 2019).

### *Kmt2e* Was Differentially Expressed Between Brain Regions and Affected by Ethanol Exposure in a Sex-Dependent Manner

**Figure 1:**
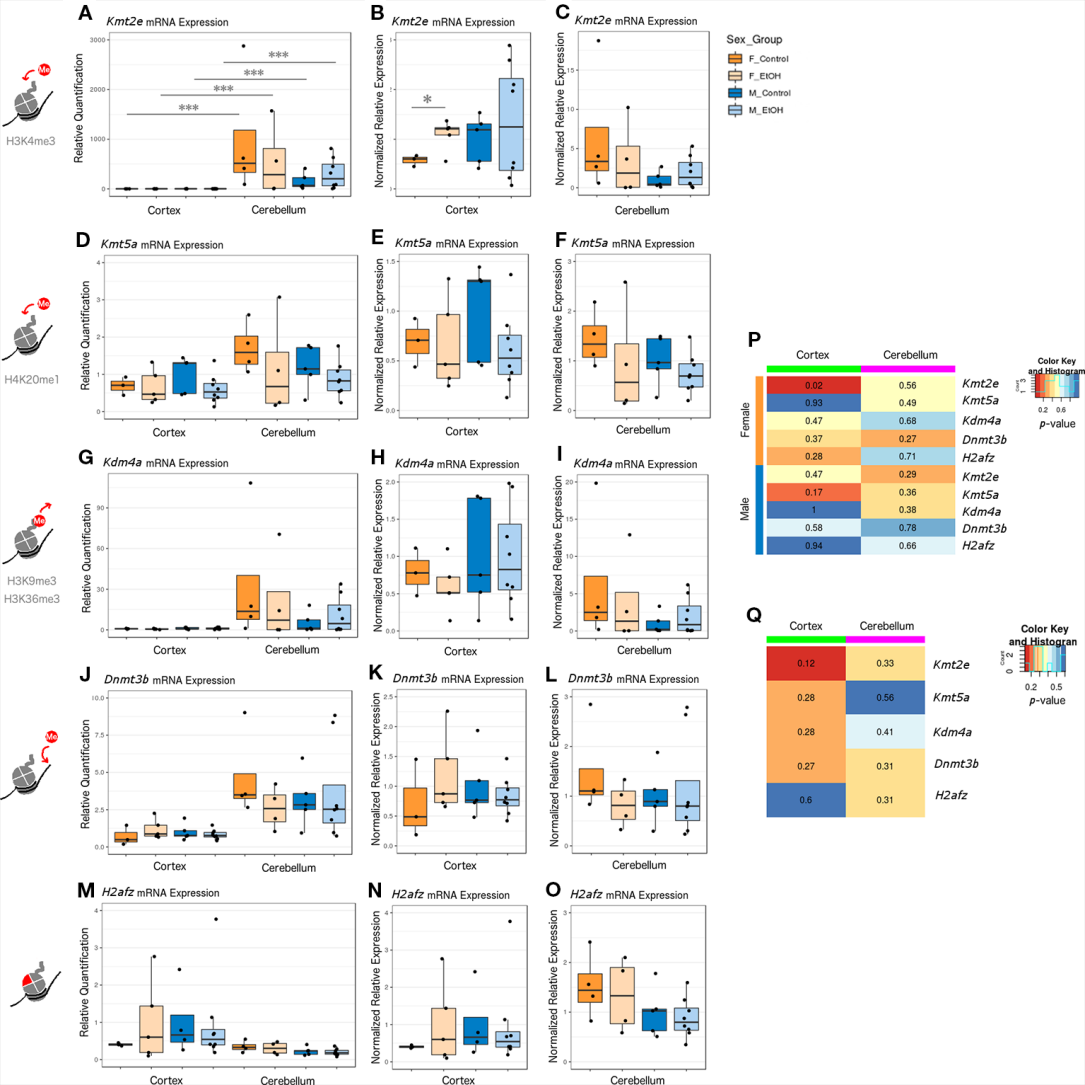
Ethanol induced differential expression of *Kmt2e* between the cortex and cerebellum and between sexes. **(A)** *Kmt2e* expression fold change over cortex of male untreated mice for each sex/brain region category. *** *p* < 0.001 (ANOVA). **(B)** *Kmt2e* expression fold change over male untreated mice in the cortex, plotted with rescaled y-axis. * *p* < 0.05 (Welch's *t*-test). **(C)** *Kmt2e* expression fold change over male untreated mice in the cerebellum. **(D)** *Kmt5a* expression fold change over cortex of male untreated mice for each sex/brain region category. **(E)** *Kmt5a* expression fold change over male untreated mice in the cortex, plotted with rescaled y-axis. **(F)** *Kmt5a* expression fold change over male untreated mice in the cerebellum. **(G–I)** *Kdm4a* expression fold change in the cortex and cerebellum of female and male mice. **(J–L)** *Dnmt3b* expression fold change. **(M–O)** *H2afz* expression fold change. **(P)** Heatmap of *p*-values from Welch's *t*-test comparing control and ethanol-treated mice for each sex/brain region category. **(Q)** Heatmap of *p*-values from Welch's *t*-test comparing male and female untreated mice for each gene.

The four chromatin modifier genes from the qPCR array and the *H2afz* gene were selected for further analysis by RT-qPCR with a larger sample size, across different brain regions, and in females. In addition to assessing effects of ethanol exposure on expression of each chromatin modifier, we directly compared the expression of each gene between the cortex and cerebellum and between sexes in all groups (**Figure 1**, female n = 8 and male n = 13). *Kmt2e*, *Kmt5a*, *Kdm4a*, and *Dnmt3b* were all expressed higher in cerebellum than cortex. This effect only reached statistical significance for *Kmt2e*, which exhibited substantial differences in the magnitude of expression between brain regions; each group of mice stratified by sex and treatment had *Kmt2e* expression up to three orders of magnitude higher in the cerebellum as compared with the cortex (**Figure 1A**, *p* < 0.001). When *Kmt2e* expression levels in the cortex were compared by sex, a trend toward lower baseline expression in female controls as compared with male controls was observed (**Figure 1B**). Interestingly, the increase in *Kmt2e* seen in the cortex of ethanol-exposed females brought mRNA levels closer to those of males at baseline (**Figure 1B**, *p* < 0.05). Baseline *Kmt2e* levels were similar between sexes in the cerebellum, though slightly higher in females (**Figure 1C**).

*Kmt5a* was also expressed slightly higher in cerebellum than cortex, on average (**Figure 1D**). No major differences between sexes at baseline in either brain region were observed (**Figures 1E, F**). There were slight trends toward reduced *Kmt5a* expression in both brain regions in ethanol-exposed animals as compared with controls, which was strongest in the male cortex (**Figures 1E, F**). While this trend was in the same direction as observed for the qPCR array (**Table S6**), ethanol-induced changes to *Kmt5a* did not reach statistical significance with this full set of samples, which suggested that only some animals were affected.

*Kdm4a* had the second-highest difference in expression between brain regions, after *Kmt2e*, and showed little variation within each brain region regarding sex or ethanol treatment (**Figures 1G–I**). The third-highest region expression difference was observed for *Dnmt3b*, which also did not show significant changes in expression regarding sex or ethanol treatment (**Figures 1J–L**). *H2afz* showed similar expression patterns between the cortex and cerebellum, with slightly more variation in cortex expression, especially in the female ethanol-treated group (**Figures 1M–O**).

Overall, the strongest evidence for differential expression with ethanol was observed for *Kmt2e* in the cortex of female mice, with an additional trend toward decreased expression of *Kmt5a* in the same brain region but in males (**Figure 1P**, *Kmt2e* female cortex *p* = 0.02 and *Kmt5a* male cortex *p* = 0.17). A trend toward sex differences in gene expression in the cortex of control mice was also observed for *Kmt2e* (**Figure 1Q**, *p* = 0.12). These results highlighted the differences in chromatin modifier expression across brain regions assessed at P7, when the cerebellum in particular experiences a growth spurt. Contrary to the qPCR array results, most of our candidate chromatin modifier genes were unaffected by ethanol exposure other than *Kmt2e* when assessed with a higher number of samples, which suggested high interindividual variability and highlighted the importance of validating results across technologies and with sufficient sample sizes.

### Ethanol Exposure Altered Bulk H3K4me3 Levels

**Figure 2:**
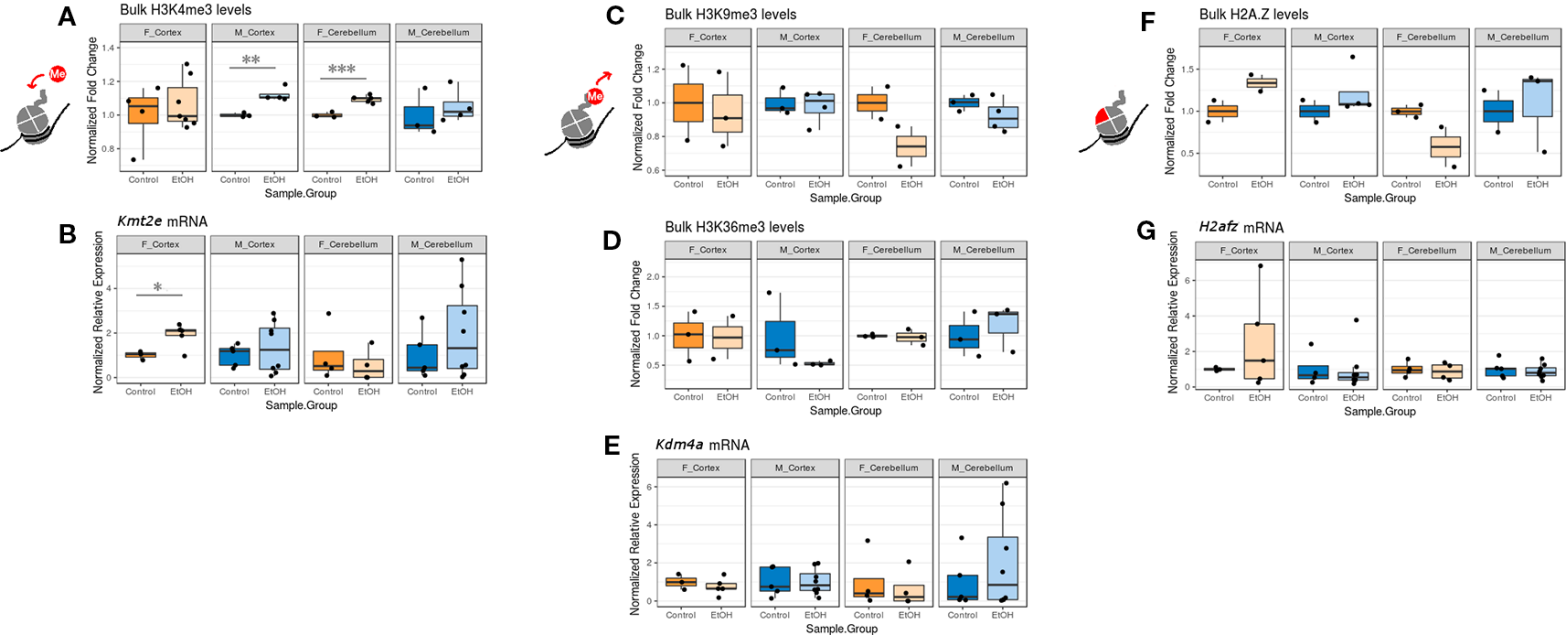
The effect of ethanol exposure on bulk histone modification/protein levels was dependent on the mark measured. **(A)** Bulk H3K4me3 immunoblot image intensity fold change over untreated mice for each sex/brain region category. ** *p* < 0.01, *** *p <* 0.001 (Welch's *t*-test). **(B)** *Kmt2e* mRNA expression fold change over untreated mice for each sex/brain region category. * *p* < 0.05 (Welch's *t*-test). **(C)** Bulk H3K9me3 immunoblot image intensity fold change over untreated mice. **(D)** Bulk H3K36me3 immunoblot image intensity fold change over untreated mice. **(E)** *Kdm4a* expression fold change over untreated mice. **(F)** Bulk H2A.Z immunoblot image intensity fold change over untreated mice. **(G)** *H2afz* expression fold change over untreated mice.

While examining expression of chromatin-modifying genes across both sexes and brain regions, we concurrently assessed whether any changes to chromatin modifier gene expression also corresponded to bulk changes in their corresponding histone PTM targets and in H2A.Z at the protein level. We focused primarily on the histone PTMs H3K4me3 (catalyzed by *Kmt2e*), H3K9me3 and H3K36me3 (both removed by *Kdm4a*), and the H2A.Z protein (encoded by *H2afz;* **Figure 2**, **Table S5**, n = 2-7 per group). Due to limited protein sample, we were only able to assess sufficient replicates for H4K20me1 (catalyzed by *Kmt5a*) protein levels in males (**Figure S1**, **Table S5**). In order to compare mRNA and protein expression, we plotted the corresponding RT-qPCR results alongside the immunoblot data for each histone modification or variant in our panel (**Figure 2**; mRNA expression results are normalized to the control group within each sex/brain region category to facilitate comparison with protein blot results).

We observed an increase in bulk H3K4me3 levels across all sexes and brain regions, which was statistically significant in the cortex of male mice and the cerebellum of female mice (*p* < 0.01 and *p* < 0.001, respectively, **Figure 2A**). This matched with the increased *Kmt2e* expression levels observed in the cortex of female ethanol-exposed mice and the cerebellum of male ethanol-exposed mice (female cortex *p* < 0.05, **Figure 2B**).

Bulk H3K9me3 and H3K36me3 levels varied little under ethanol exposure across groups, although a trend toward decreased H3K9me3 in the cerebellum of both sexes was noted (**Figures 2C, D**). These results were similar to the *Kdm4a* mRNA expression results, which also showed little change associated with ethanol (**Figure 2E**).

Ethanol exposure induced a trend in bulk H2A.Z protein levels; ethanol-exposed females exhibited higher H2A.Z levels in the cortex, and ethanol-exposed males exhibited higher H2A.Z levels in both the cortex and cerebellum. Female alcohol-treated animals, conversely, had lower H2A.Z in the cerebellum (**Figure 2F**). This was in contrast to the *H2afz* mRNA expression levels, which were not significantly altered with ethanol exposure, though ethanol-exposed female mice had more variable levels of *H2afz* in the cortex (**Figure 2G**).

Overall, the correlation between mRNA expression and protein varied depending on the mark examined. Our results provided some evidence that ethanol exposure may be dysregulating the epigenetic machinery that is responsible for catalyzing histone PTMs, in the case of *Kmt2e* and H3K4me3.

### Ethanol Induced Limited Alterations to DNA Methylation Levels At the *Kdm4a* Promoter in the Cerebellum of Female Mice

In order to assess upstream regulatory changes associated with altered chromatin-modifying gene expression, we evaluated *Kdm4a* and *Kmt2e* promoter DNA methylation levels by bisulfite pyrosequencing (**Table S3**).

Thirteen CpG sites were analyzed across the *Kmt2e* promoter (**Tables S4** and **S5**, **Figure S2**, n = 2–9 per group). No statistically significant differences in DNA methylation between ethanol and control animals were found for either sex in the cortex or cerebellum, and all effect sizes were smaller than 5%, within the technical error rate of our bisulfite pyrosequencing assay (**Figure S2**).

**Figure 3:**
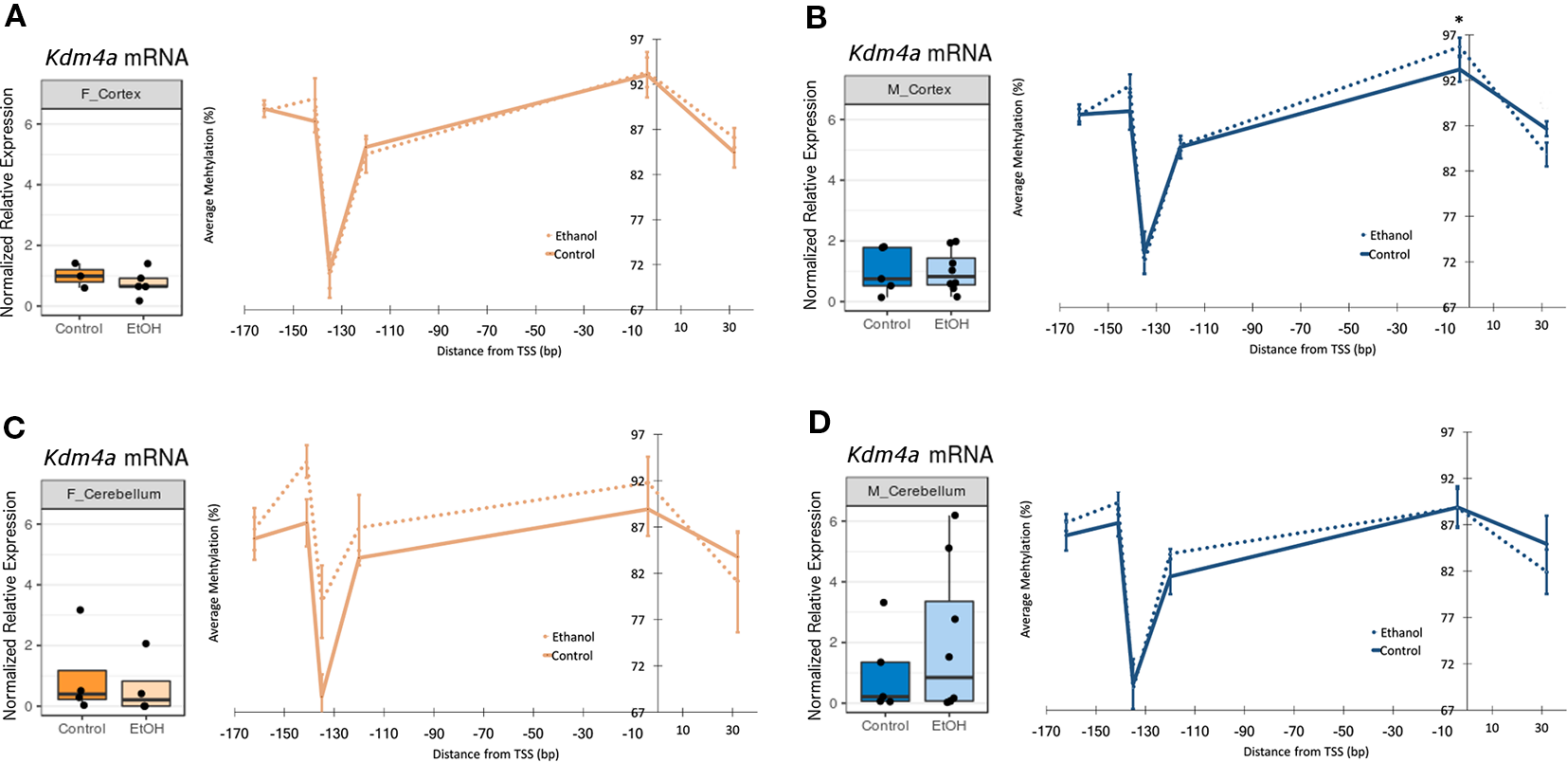
Ethanol induced a trend toward altered *Kdm4a* promoter DNA methylation in the female cerebellum. Fold change in expression over untreated mice for each sex/brain region category and percent methylation of *Kdm4a* promoter for: **(A)** female cortex; **(B)** male cortex, * *p* < 0.05 (Welch's *t*-test); **(C)** female cerebellum **(D)** male cerebellum. Error bars: SEM.

Six CpG sites were assessed across the *Kdm4a* promoter (**Tables S4** and **S5**, **Figure 3**, n = 4-10 per group). In the cortex of female mice, no ethanol-induced changes in DNA methylation were observed (**Figure 3A**). In the cortex of males, ethanol did not induce any changes in DNA methylation with a magnitude above 5% (**Figure 3B**). The cerebellum of female ethanol-exposed animals showed the largest effect size, with a maximum 10% increase in DNA methylation at the −135 site, and a mean 3.5% increase in DNA methylation across the entire promoter region; however, this was not statistically significant, likely due to high interindividual variability (**Figure 3C**). The cerebellum of male animals did not display any notable changes in DNA methylation (**Figure 3D**). Overall, the few changes to *Kdm4a* promoter DNA methylation in the cortex of both sexes and cerebellum of males were consistent with the similar *Kdm4a* mRNA expression levels between control and ethanol-exposed mice. The larger increase in DNA methylation in the cerebellum of females may correlate with a slight trend toward decreased *Kdm4a* expression in the ethanol-exposed group, though this will require replication with a larger sample size.

### Ethanol Altered *Casp6 and Ltbr* Expression in the Cortex and Cerebellum of a Subset of Animals

After exploring the effects of ethanol on chromatin modifiers (involved in cell proliferation and differentiation, among other processes), we next examined whether neonatal ethanol exposure affected expression of apoptotic genes in our mice. While chromatin modifiers influence neurogenesis and differentiation in the developing brain, apoptotic genes impact the rates of cell death, which is also tightly regulated in typical development and has been previously shown to be dysregulated upon ethanol exposure (Subbanna et al., 2013; Subbanna et al., 2014).

Apoptosis can occur through several pathways each involving many intermediates, and at least 70 genes have been implicated in cortical and cerebellar neuronal apoptosis specifically (Cavarallo et al., 2004; Elmore, 2007). To identify candidate genes underlying apoptotic pathways in our model, we again used a SABiosciences qPCR array panel to assay the expression of 84 apoptotic genes in the cortex and cerebellum of male mice (n = 3 per group for cortex and n = 2–3 per group for cerebellum; **Tables S7** and **S8**, **Figure S5**). We selected the lymphotoxin-beta receptor (*Ltbr*) gene for RT-qPCR follow-up due to its trend toward significance in both brain regions (cortex FC = 1.60 and *p* = 0.098; cerebellum FC = 1.23 and *p* = 0.017). We also included the caspase-6 (*Casp6*) gene in our RT-qPCR panel, due to its high significance in cortex and our confidence in the larger sample size in this brain region (cortex FC = 1.29 and *p* = 0.001; cerebellum FC = 1.60 and *p* = 0.22).

**Figure 4:**
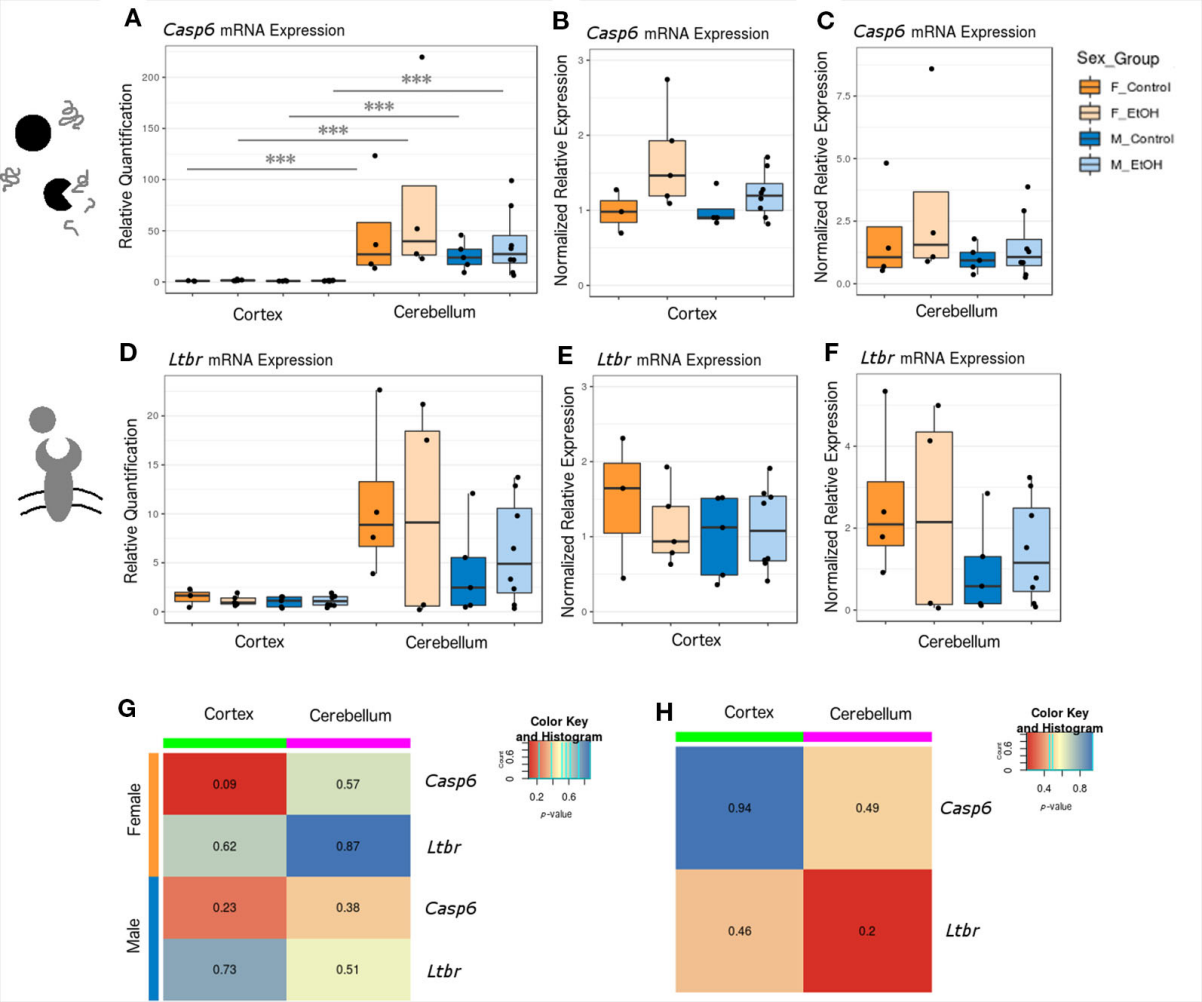
Ethanol exposure induced trends toward elevated *Casp6* expression and differential expression of *Casp6* and *Ltbr* between brain regions. **(A)** *Casp6* expression fold change over cortex of male untreated mice for each sex/brain region category. *** *p* < 0.001 (ANOVA). **(B)** *Casp6* expression fold change over male untreated mice in the cortex, plotted with rescaled y-axis. **(C)** *Casp6* expression fold change over male untreated mice in the cerebellum. **(D)** *Ltbr* expression fold change over cortex of male untreated mice for each sex/brain region category. **(E)** *Ltbr* expression fold change over male untreated mice in the cortex, plotted with rescaled y-axis. **(F)** *Ltbr* expression fold change over male untreated mice in the cerebellum. **(G)** Heatmap of *p*-values from Welch's t test comparing control and ethanol-treated mice for each sex/brain region category. **(H)** Heatmap of *p*-values from Welch's *t*-test comparing male and female untreated mice for each gene.

*Casp6* and *Ltbr* were examined across both brain regions in a larger set of animals, including females, using RT-qPCR (**Figure 4**, female n = 8 and male n = 13). *Casp6* was more highly expressed in cerebellum than in cortex for each sex and treatment group compared (**Figure 4A**, *p* < 0.001). When comparing untreated versus ethanol-exposed mice, a trend emerged toward increased *Casp6* expression with ethanol exposure regardless of sex or brain region (**Figures 4B, C**). *Ltbr* also had a trend toward increased expression in cerebellum (**Figures 4D–F**). No significant differences in *Ltbr* expression upon ethanol exposure were identified, although there was a trend toward higher expression in female mice at baseline in the cerebellum (**Figures 4D–H**, *p* = 0.20). If the trend toward altered *Casp6* mRNA levels could be supported by additional evidence in future studies (such as caspase cleavage assays, TUNEL assays, or similar), this may shed light on whether caspase-mediated apoptotic signaling is activated across the cortex and cerebellum of animals exposed to ethanol at P7.

### Neonatal Alcohol Exposure Increased γH2A.X Levels in the Cortex

We next examined whether changes in apoptotic gene expression corresponded to altered levels of histone PTMs related to apoptotic pathway activation. This would allow us to better determine to what degree apoptotic signaling cascades may have been activated in our animals. Bulk levels of γH2A.X, a marker of DNA damage, and H2B-S14P, which is induced by caspase-mediated apoptotic signaling, were assessed by protein blotting (Fernandez-Capetillo et al., 2004).

**Figure 5:**
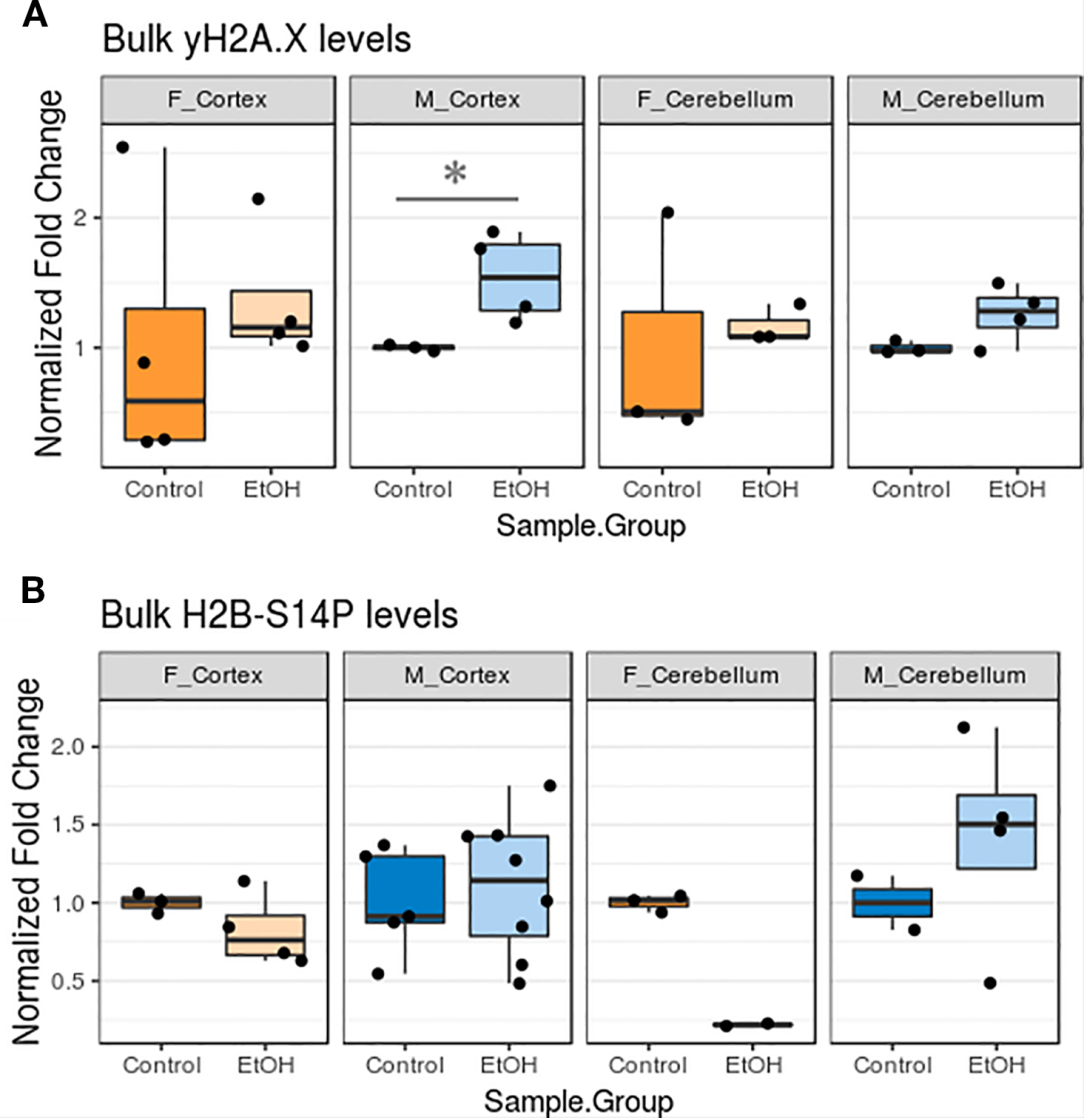
Ethanol increased bulk γH2A.X levels and had sexually dimorphic effects on bulk H2B-S14P levels in the cortex and cerebellum. **(A)** Bulk γH2A.X immunoblot image intensity fold change over untreated mice for each sex/brain region category. * *p* < 0.05 (Welch's *t*-test). **(B)** Bulk H2B-S14P immunoblot image intensity fold change over untreated mice for each sex/brain region category.

Ethanol exposure elevated γH2A.X levels in all animals, while effects on H2B-S14P levels varied (**Figure 5**, **Table S5**, n = 2–8 per group). The γH2A.X increase was most robust in the cortex of male animals (*p* < 0.05, **Figure 5A**). Unexpectedly, trends in H2B-S14P changes upon ethanol exposure were different according to sex. H2B-S14P levels were lower in the cortex and cerebellum of female mice exposed to alcohol, while levels of this mark either stayed the same or increased in males (**Figure 5B**).

### Ethanol Exposure Did Not Induce DNA Methylation Changes At the *Ltbr* Promoter

After observing ethanol-induced changes to gene expression of *Casp6* and *Ltbr* in our initial qPCR array investigation, we evaluated whether these gene expression changes could be driven by changes to promoter DNA methylation. We designed a pyrosequencing assay for *Ltbr* targeting three CpGs across the promoter region (**Tables S3–S5**, n = 5–10 per group). Minor DNA methylation changes were observed in the cortex and cerebellum; however, none of these were statistically significant (**Figure S3**). This was not surprising, given that *Ltbr* expression did not vary much between ethanol-exposed and control animals when examined by RT-qPCR.

## Discussion

**Figure 6:**
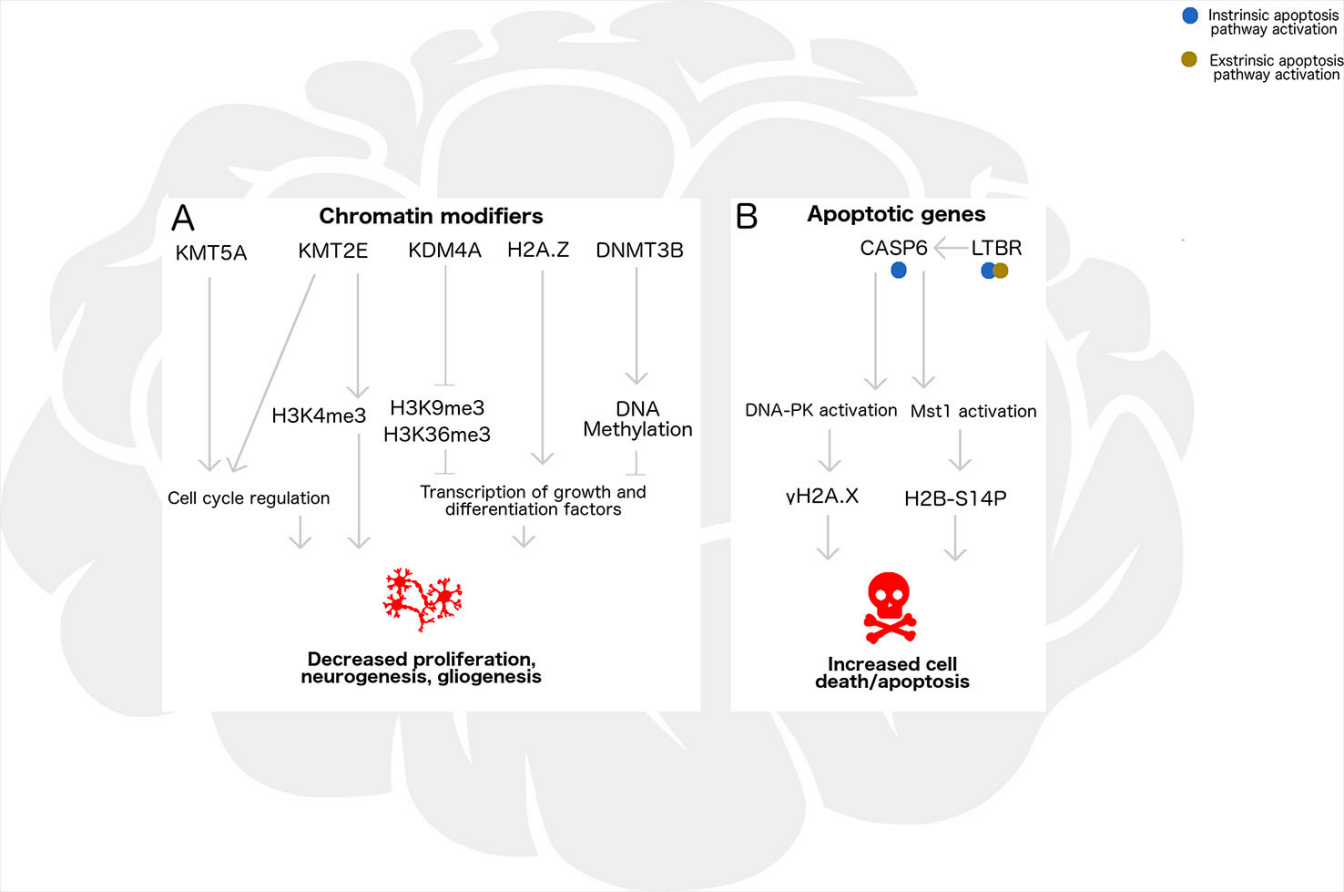
Epigenetic regulation by chromatin remodelers and histone posttranslational modifications may influence neurogenesis/gliogenesis, cell cycle progression, transcription, and apoptosis in the developing ethanol-exposed brain. **(A)** In the developing brain, chromatin modifying enzymes Kmt5a, Kmt2e, and Kdm4a catalyze addition or removal of histone posttranslational modifications which influence neuronal differentiation and S phase progression in the mitotic cell cycle. Additionally, H2A/H2A.Z exchange and DNA methylation catalyzed by Dnmt3b may be associated with transcriptional regulation of genes underlying these pathways, such as repressors, transcription factors, and chromatin remodelers. Under binge ethanol exposure conditions, dysregulation of chromatin modifying enzymes could lead to decreased cellular proliferation, neurogenesis, and gliogenesis in the developing brain. **(B)** Apoptosis may also be triggered by ethanol exposure through intrinsic or extrinsic pathways, influencing transcription of *Casp6* and *Ltbr*. Casp6 activation and subsequent DNA-PK and Mst1 activation lead to formation of histone modifications gH2A.X and H2B-S14P, which take part in signaling that could lead to increased cell death under ethanol exposure.

In this study, we assessed gene expression, histone modifications, histone variant levels, and promoter DNA methylation in the cortex and cerebellum of female and male neonatal mice following acute alcohol exposure. In **Figure 6**, we propose a model encompassing the regulation of neurogenesis/gliogenesis and cell death in the developing neonatal brain by the epigenetic factors investigated in this work. While ethanol induced few overall epigenomic changes, exposed mice had altered expression of the two genes most highly expressed in cerebellum in a similar manner across brain regions and sexes, and also exhibited trends toward altered histone PTMs associated with these genes. *Kmt2e* was expressed up to several thousand-fold higher in cerebellum than cortex, and a trend toward increased *Kmt2e* levels with ethanol exposure accompanying elevated levels of bulk H3K4me3 was observed. Similarly, *Casp6* was expressed up to two orders of magnitude higher in cerebellum than in cortex, and a pattern of elevated *Casp6* expression upon ethanol exposure was observed for all mice, accompanied by increased γH2A.X levels.

Some of the strongest findings in our study were the differences in *Kmt2e* and *Casp6* expression both across brain regions and within control-ethanol group comparisons. We found that both genes were expressed higher in the cerebellum of neonatal mice than the cortex, consistent with expression patterns that have been observed in human brain tissue (Carithers et al., 2015). Expression of these genes may be particularly crucial at the postnatal day 7 stage when these mice were exposed to ethanol. During this “critical period” of brain development, spurts of neurogenesis and gliogenesis are occurring (Dobbing and Sands, 1974). The cerebellum in particular continues to develop postnatally, with specialized cell types forming up until postnatal day 21 in mice (Butts et al., 2014). During this period, H3K4me3 patterns which influence transcription and pluripotency/differentiation are extensively remodeled, and programmed cell death also occurs in a highly controlled manner (**Figures 6A, B**, Shen et al., 2014; Yamaguchi and Miura, 2015). By altering expression of these genes and levels of histone PTMs regulating proliferation and apoptosis, ethanol exposure could disrupt the balance between neurogenesis/gliogenesis and cell death in the developing brain.

Ethanol exposure has previously been associated with global histone PTM alterations, particularly H3K4me3; however, our results are in contrast to several studies which reported reductions in brain H3K4me3 in response to alcohol (Veazey et al., 2013; Gavin et al., 2018; Jarmasz et al., 2019). These findings were reported in human postmortem brain, rat brain, and neural stem cells, respectively, and did not assess the cerebellum. Additionally, the timing of ethanol exposure and tissue harvesting varied across studies, from 72 h of ethanol vapour treatment in adult rats, to 5 days of treatment in neural stem cells, to varied amounts of exposure among a cohort of prenatally exposed infants (Veazey et al., 2013; Gavin et al., 2018; Jarmasz et al., 2019). It is possible that the impact of ethanol on H3K4me3 levels may differ by length and timing of exposure, since our study assessed rapid epigenetic changes observable 7 h after ethanol treatment. The effect of ethanol on global H3K4me3 may also vary by species/strain and tissue, and future studies should continue to assess directionality of ethanol-induced global H3K4me3 changes.

Although our combined histone PTM and chromatin modifier gene expression results did not agree with each other overall, some specific cases stand out. In particular, *Kmt2e* and H3K4me3 levels both trended toward an increase in the cortex of some ethanol-exposed animals. However, the *Kmt2e* expression increase only reached significance in females, while the H3K4me3 increase only reached significance in males. Several factors may explain this pattern. For example, other MLL-family histone methyltransferases can catalyze formation of H3K4me3, levels of which may have been influenced in this study (Ruthenburg et al., 2007). Additionally, since limited sample volume did not permit us to measure protein expression of Kmt2e, we cannot be certain that *Kmt2e* mRNA expression levels reflect the level of active enzyme and are directly related to altered H3K4me3 patterns. There may also be a temporal lag between change in *Kmt2e* mRNA and production of Kmt2e protein, which we are unable to detect in this cross-sectional analysis. Future studies should examine these marks with a larger sample size and assess Kmt2e protein in addition to mRNA.

While this is not the first study to report altered H3K4me3 levels upon ethanol exposure, to our knowledge, previous work has not explored the relationship between H3K4me3 and *Kmt2e* expression under these conditions. While we observed few ethanol-related differences in expression of *Kmt5a*, *Kdm4a*, *Dnmt3b*, and *H2afz*, some interesting trends in their corresponding histone PTMs and encoded protein emerged, including reduced H3K9me3 and H2A.Z in female cerebellum and increased H2A.Z in the cortex and in male cerebellum. To our knowledge, H3K9me3 and H2A.Z have not been comprehensively assessed in the context of neonatal alcohol exposure either apart from initial studies on hippocampal H2A.Z and H2A.Z turnover (Rachadaoui et al., 2017; Gretzinger et al., 2019). Although these trends were interesting, it should be noted that limited samples were available for H2A.Z quantification, and this finding should be replicated to determine statistical robustness. Future studies should examine whether these ethanol-associated alterations to H3K4me3, H3K9me3, and H2A.Z levels correlate with any deficits in neonatal brain development.

The small sample sizes used in this study may also partially explain some of the small effects observed for *Kmt2e, Kdm4a*, and *Ltbr* promoter DNA methylation. While some site-specific changes in *Kdm4a* promoter DNA methylation in male cortex were statistically significant, our confidence in detecting the small magnitude and the biological relevance of this is unclear. Small magnitude effect sizes in DNA methylation can have an impact on transcriptional activity, but these should be considered in the context of the accuracy of the measurement platform (Breton et al., 2017). The larger change in *Kdm4a* promoter DNA methylation in the female cerebellum is more likely to be meaningful, since it also associated with the trend toward decreased *Kdm4a* expression in this sex and brain region. This likely did not reach statistical significance due to interindividual variability and/or cell-type heterogeneity within the sample.

The unique patterns of increased *Kdm4a* promoter DNA methylation in female cerebellum, reduced H3K9me3 and H2A.Z in the female ethanol-exposed cerebellum, and reduced H2B-S14P in female cortex and cerebellum suggested some level of sexual dimorphism was present in ethanol response.

This could be the result of sex chromosome gene dosage, or differing gonadal hormone levels (Schulz and Sisk, 2016). Either way, our study suggested that the cerebellum could be particularly vulnerable to sex differences in chromatin modifier and apoptotic responses to neonatal binge ethanol exposure. It is tempting to speculate that this may translate to phenotypic differences: deficits in sensory and motor processes regulated by the cerebellum have been demonstrated in children with FASD, and sexual dimorphisms have been observed in cerebellar-driven sensory circuits of adults with FASD (Tesche et al., 2014; Paolozza et al., 2015; Hoyme et al., 2016).

The neurodevelopmental disabilities associated with FASD may also be due in part to increased rates of neuronal cell death, a phenomenon which has been well demonstrated in animal models of developmental alcohol exposure (**Figure 6B**; Subbanna et al., 2013; Goldowitz et al., 2014; Subbanna et al., 2014). The observations of ubiquitously increased *Casp6* expression and γH2A.X levels following ethanol exposure in our model agree with this previous literature, suggesting that ethanol could have induced DNA double strand breaks and perhaps activated caspase-mediated apoptotic signaling. Future studies should continue examining this, and directly assess whether double-stranded breaks and apoptotic signaling cascades are activated in the P7 brain under acute alcohol exposure. Caspase-6 activation has been demonstrated in response to apoptotic signals, and overexpression of *Casp6* renders cells more vulnerable to apoptotic death, which may also be the case in our model (MacLachlan and El-Deiry, 2002).

Perhaps unexpectedly, a sex difference in H2B-S14P trends emerged, with lowered H2B-S14P levels observed in both the cortex and cerebellum of ethanol-exposed females and higher H2B-S14P in the cortex and cerebellum of ethanol-exposed males. The effect of this discrepancy on cell death is unclear, since H2B-S14P has been previously reported to be both necessary and dispensable for apoptosis (Wong et al., 2009; Ajiro et al., 2010). However, sexual dimorphisms in cell death mechanisms have previously been demonstrated in both cortical and cerebellar neurons before; for example, XY cortical neurons respond to cytotoxic challenge primarily through apoptosis, while XX neurons respond *via* a cytochrome c-dependent pathway (Du et al., 2004; Sharma et al., 2011). It is possible that the rate and/or mechanism of cell death could be different in females, and future studies should continue exploring this phenomenon.

Despite intriguing trends in specific histone PTMs and expression of a select few genes, ethanol induced few overall changes to the epigenetic and transcriptional marks examined in this study. It is possible that the effects of alcohol exposure on the neonatal mouse brain after 7 h may be subtle, as some studies have demonstrated stronger epigenetic and transcriptional responses to ethanol given a longer exposure paradigm (Guo et al., 2011; Lussier et al., 2015). Factors such as cell-type heterogeneity and limited sample sizes may additionally make smaller short-term effects of ethanol difficult to detect. Nevertheless, our results highlighted that ethanol could have either no impact or a primarily subtle impact on chromatin modifier and apoptotic genes in the cortex and cerebellum at the P7 stage.

Although this study provided an overall survey of early chromatin and apoptotic interactions upon alcohol exposure, several limitations should be taken into consideration. First, we did not assess female samples by qPCR array as part of the initial candidate gene discovery, which could have yielded different results. We intended to find genes differentially expressed in both sexes and thus selected one sex for discovery, with males matching the majority of the existing literature body on PAE. However, it would be pertinent for future experiments to examine females and males concurrently from the start. Second, some of the effects observed were small in magnitude and/or noisy. Several factors may explain this high variability, such as differences in cell-type proportions across samples, interindividual variation between mice, and constraints of sample availability for adequate statistical power to confidently detect small differences. Third, histone modifications were measured in bulk, which lowers the ability to detect potentially important site-specific changes. Several genome-wide surveys of histone marks have already been performed in rodent and cell culture systems, providing a baseline for site-specific reference. For example, previous work in alcohol-exposed mice and neurospheres has demonstrated altered H3K4me3 patterns specifically at promoters of genes involved in neuronal identity and differentiation; this could also be the case in our model (Shulha et al., 2013; Veazey et al., 2013; Veazey et al., 2015). Finally, the animal model in our study reflects an acute, high-dose ethanol exposure. While this exposure paradigm may not be reflective of chronic or long-term alcohol use during pregnancy, it allowed us to investigate epigenetic changes in the brain which can occur rapidly following alcohol exposure.

## Conclusions

In summary, we identified global changes in H3K4me3 and caspase-driven apoptotic marks upon ethanol exposure that were consistent between sexes and across the cortex and cerebellum. We additionally observed trends that could indicate sexually dimorphic effects of ethanol on H3K9me3, H2A.Z, and H2B-S14P levels, as well as *Kdm4a* promoter DNA methylation. We propose that while ethanol has few overall effects on expression of chromatin modifier and apoptotic genes in our paradigm, this substance can modify expression of certain genes and histone PTMs rapidly following binge exposure. We also suggest that major ethanol-associated changes to chromatin persist between sexes and brain regions, and that minor changes to chromatin modifiers and apoptotic signaling may display some sexual dimorphism.

This study provided additional evidence that ethanol exposure may be altering regulation of epigenetic modifiers responsible for histone PTM addition and removal. We also presented data for the impact of ethanol on H2A.Z levels in cortex and cerebellum, which was previously reported in hippocampus (Gretzinger et al., 2019). Our findings are consistent with the need to consider the effects of ethanol exposure on gene expression in the context of brain region and sex, in order to evaluate whether ethanol-induced changes to expression are within an expected range for sex and tissue. These results bring us one step closer to elucidating the molecular pathogenesis of FASD, as well as epigenetic regulation and sex differences in behavioral outcomes.

The implications of this study reach far beyond PAE, highlighting the sensitivity of the developing brain, and that factors such as neonatal environment and sex-specific influences may act through epigenetic regulation. Profiling early genetic, epigenetic, and transcriptional responses to environmental insults will clarify the pathogenesis of other developmental disorders and potentially lead to prevention and treatment options. The era of new genetic technologies offers exciting opportunities in neurodevelopmental research for improving the lives of children and adults with FASD.

## Data Availability Statement

The raw data supporting the conclusions of this article will be made available by the authors, without undue reservation, to any qualified researcher.

## Ethics Statement

The animal study was reviewed and approved by Institutional Animal Care and Use Committee, UTHSC.

## Author Contributions

KH, JB, DG, MK, and AL designed the study. JB and KH performed the animal work, including ethanol injections and dissections. AL extracted histones and RNA from male cortex and cerebellum samples, and performed Western Blot and qPCR array analysis. SS extracted RNA and DNA from all remaining samples, performed qPCR, pyrosequencing, and additional Western Blot analysis, and wrote the manuscript. All authors read and approved the final manuscript.

## Funding

SS was supported by a Faculty of Medicine Graduate Award from the University of British Columbia. AL was supported by a Developmental Neurosciences Research Training Award from Brain Canada and the Kid's Brain Health Network (formerly NeuroDevNet). JB and KH were supported by the National Institute on Alcohol Abuse and Alcoholism (NIAAA: R01AA023508). MK is the Canada Research Chair in Social Epigenetics, Senior Fellow of the Canadian Institute for Advanced Research, and Sunny Hill BC Leadership Chair in Child Development.

## Conflict of Interest

The authors declare that the research was conducted in the absence of any commercial or financial relationships that could be construed as a potential conflict of interest.
