# Supplementary material for: Neonatal Alcohol Exposure in Mice Induces Select Differentiation- and Apoptosis-Related Chromatin Changes Both Independent of and Dependent on Sex
Source: Front Genet. 2020 Feb 11;11:35. doi: 10.3389/fgene.2020.00035 (PMC7026456; doi:10.3389/fgene.2020.00035)
Supplement: Supplementary file 1 [file DataSheet_1.pdf]

Supplementary Material

1 Supplementary Tables

Table S1. Mouse Litter Information

Name	Treatment Date	Number of Mice		
		Male	Female	Total
082112	Aug 21, 2012	3	2	5
022113	Feb 21, 2013	3	2	5
022413	Feb 24, 2013	4	2	6
031814	Mar 18, 2014	3	3	6
032014	Mar 20, 2014	3	2	5

Table S2. Antibodies used for Immunoblotting

Dilution	Origin	Target	Cat no./Vendor
1/1,000	Rabbit polyclonal	Histone H2A.X	ab10475, abcam
1/1,000	Mouse monoclonal	Histone H2A.X (phospho-Ser139)	ab18311, abcam
1/2,000	Rabbit polyclonal	Histone H2A.Z	ab4174, abcam
1/2,000	Mouse monoclonal	Histone H3	ab10799, abcam
1/2,000	Rabbit polyclonal	Histone H3	ab1791, abcam
1/2,000	Rabbit polyclonal	Histone H3 (acetyl Lys9)	ab4441, abcam
1/2,000	Rabbit polyclonal	Histone H3 (acetyl Lys14)	06-911, Millipore
1/2,000	Rabbit polyclonal	Histone H3 (mono-methyl Lys4)	ab889, abcam
1/2,000	Rabbit monoclonal	Histone H3 (di-methyl Lys4)	ab32356, abcam
1/2,000	Rabbit polyclonal	Histone H3 (tri-methyl Lys4)	ab8580, abcam
1/5,000	Rabbit polyclonal	Histone H3 (tri-methyl Lys9)	AR-0170-50, LPBIO
1/2,000	Rabbit polyclonal	Histone H3 (di-methyl Lys27)	ab24684, abcam
1/2,000	Mouse monoclonal	Histone H3 (tri-methyl Lys27)	ab6147, abcam
1/5,000	Rabbit polyclonal	Histone H3 (tri-methyl Lys36)	ab9050, abcam

1/1,000	Mouse monoclonal	Histone H4	ab31827, abcam
1/5,000	Rabbit polyclonal	Histone H4 (acetyl)	06-866, Millipore
1/2,000	Rabbit polyclonal	Histone H2B	07-371, Millipore
1/1,000	Mouse monoclonal	Histone H2B (phospho-Ser14)	61011, Active Motif
1/10,000	IRDye® 800CW conjugated goat polyclonal	Mouse IgG	926-32210, Li-Cor Biosciences
1/15,000	IRDye® 680 conjugated goat polyclonal	Rabbit IgG	926-32221, Li-Cor Biosciences

Table S3. Primer Sequences

Assay	Primer name	Sequence (5’ → 3’)	Product size (bp)
qPCR	Casp6_qPCR_F	CCTCCCCTTTTGTGACACTG	166
	Casp6_qPCR_R	ACCAAGTCAAATAGGCCCACTA	
	Ltbr_qPCR_F	AACCTATTTGCCACCTGTGC	179
	Ltbr_qPCR_R	GCTAGTGTCCGGTTCTGCTC	
	Kmt2e_qPCR_F	TCCCCCTCCTTCATCCTACT	173
	Kmt2e_qPCR_R	GCCCTGGAGTAACGTGATGT	
	Kdm4a_qPCR_F	CAAGCCTATTGTGGGTGCTT	206
	Kdm4a_qPCR_R	TACAAAAGGGGATTGGGTCA	
	Dnmt3b_qPCR_F	GGGGCATCACTGGAGATAAG	201
	Dnmt3b_qPCR_R	CTCCCCACACCTGTGAAGTT	
	Setd8_qPCR_F	CTTGAAGCATCAGGGTGGAC	249
	Setd8_qPCR_R	AACCGCATTCATTTCACTCC	
	H2Afz_qPCR_F	TTGGATTTTAATTGTGATGCAGA	173

	H2Afz_qPCR_R	GCAGAGAATGTACTTGTTGCTGAG	
Pyrosequencing	Ltbr_A1_F1	GGTTGTTATTGTAGGTGTGTAGAATAT	369
	Ltbr_A1_R1	ACCTTCAAAAACCTTAAAAACTCATATTAC*	
	Ltbr_A1_S1	GTGGGGTGTTGTTTG	n/a
	Kmt2e_A1_F1	GGGGATAAAGGGTAAAGAGTTAAGTT	332
	Kmt2e_A1_R1	AAACTACTCCCAATATCTATACCCCCTCTA*	
	Kmt2e_A1_S1	AGTTAAGTTTATTAGTTAGTAAGATTT	n/a
	Kmt2e_A1_S2	GAAAGAAGGAGTGTTTGGTTA	
	Kdm4a_A1_F1	GATGATATTAAAAGGGGTTTAAGTGAAAT	353
	Kdm4a_A1_R1	CCCAATATAAAAAACTTCATACCTTACTC*	
	Kdm4a_A1_S1	AGTTATAATGTTTTGGATGT	n/a
	Kdm4a_A1_S2	TTGTTTAATTTATTTTATTGTTAAGGAT	

**5' biotin tag*

Table S4. Pyrosequencing Target Regions

Primer name	Sequence to analyze (5' → 3')	Length (bp)
Ltbr_A1_S1	AATTTGGAAGTYGGGATTTTTTYGGAAGYGTTTTTGAGG GTTTTGTGTTTTTATTT	56
Kmt2e_A1_S1	TTTTAATYGTYGGYGGATGGATGAATGTATTTGTTGTGG GAAAGAYGGAGTGTTTGGTTAGTT	73
Kmt2e_A1_S2	GTTYGYGGTTTTTAGYGTGAYGTTTTTAYGTTTYGGYGG AGAGGTYGYGT	75

Kdm4a_A1_S1	TTAAYGGGTTATAGTATTTATTTTYGTGAAYGGTGTTTAT TTTAGYGAATAGTTTTTTAGTTTTTGTTTTATA	63
Kdm4a_A1_S2	AAGGTAATGTATYGGTTTTTAAAATAATTTGGTTTATAG AGTTTATAYGTAATAAAAAAGGGGAAGTATTATAAA	50

Table S5. Number of samples used for experiments.

	**Cortex**				**Cerebellum**			
	**Female**		**Male**		**Female**		**Male**	
**Measure**	**Control**	**Ethanol**	**Control**	**Ethanol**	**Control**	**Ethanol**	**Control**	**Ethanol**
mRNA expression (all)	3	5	5	8	4	4	5	8
H3K4me3	4	7	3	4	4	5	3	4
H3K9me3	2	3	3	4	2	2	3	4
H3K36me3	3	2	3	3	3	3	3	3
H4K20me1	1	2	3	3	1	1	3	3
H2A.Z protein	2	2	2	4	3	2	2	3
Kmt2e DNA methylation	2	3	4	6	5	5	6	9
Kdm4a DNA methylation	4	5	6	10	5	8	6	8
Ltbr DNA methylation	5	5	6	10	5	5	6	9
γH2A.X	4	4	3	4	3	3	3	4
H2B-S14P	3	4	5	8	3	2	2	4

2 Supplementary Figures

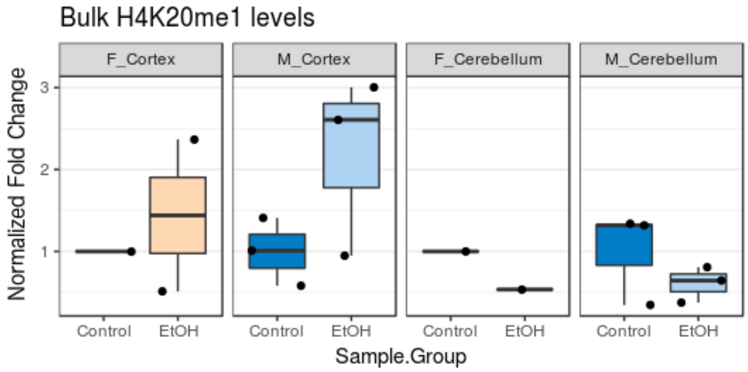

Supplementary Figure 1. Ethanol had sexually dimorphic effects on H4K20me1 levels in the cortex and cerebellum. Fold change in immunoblot image intensity over control of histone modifications for H4K20me1.

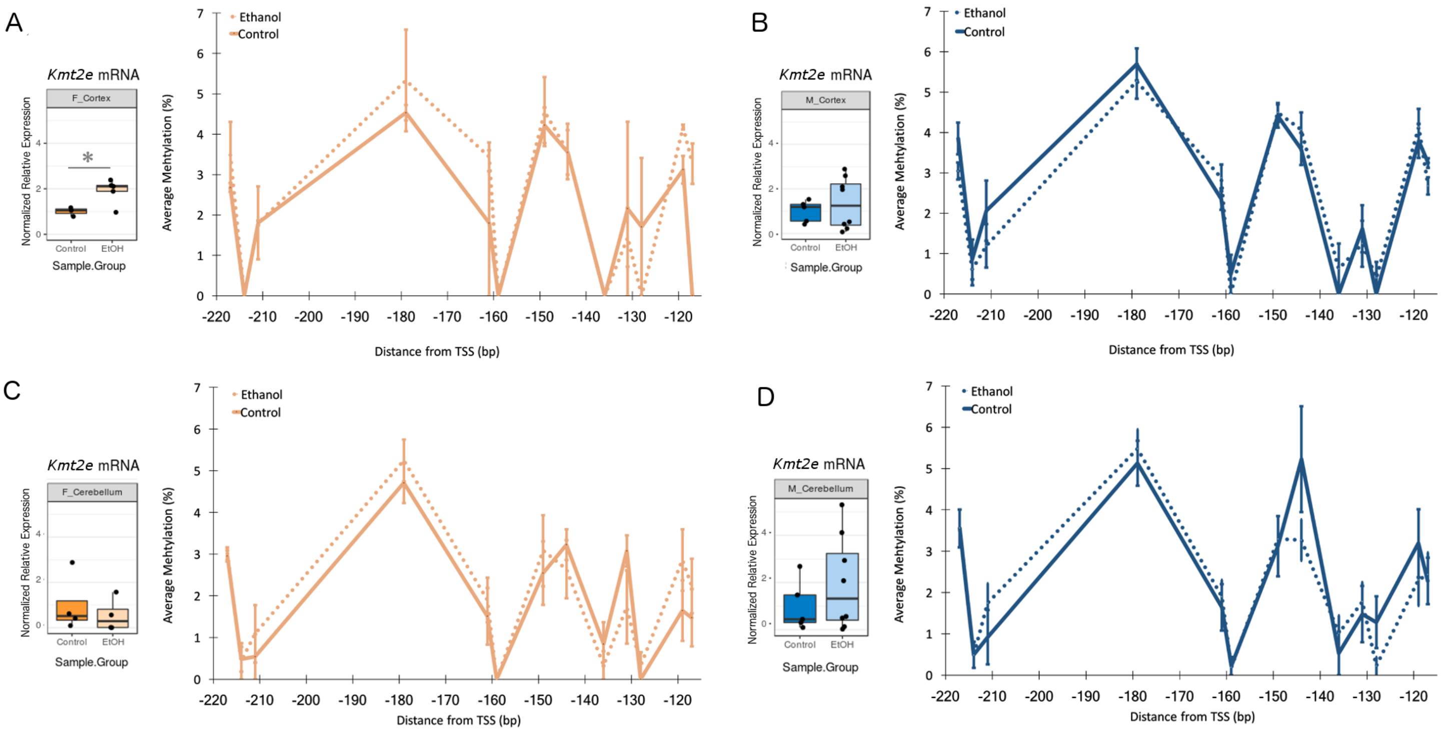

Supplementary Figure 2. Ethanol did not alter *Kmt2e* promoter DNA methylation. Fold change in expression over *Pgk1* and percent methylation of *Kmt2e* promoter for: **(A)** female cortex; **(B)** male cortex; **(C)** female cerebellum; **(D)** male cerebellum. Dark blue: male; pale orange: female. Error bars: SEM.

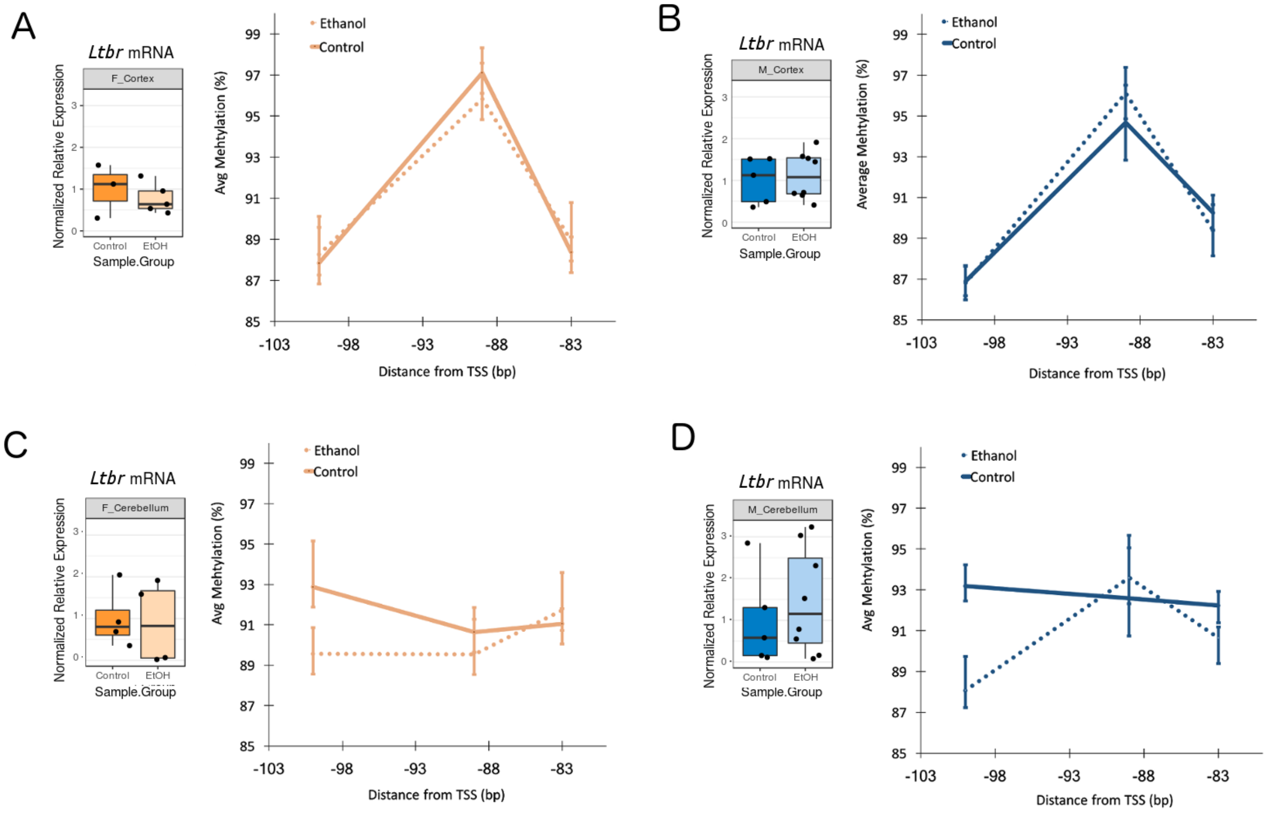

Supplementary Figure 3. Ethanol-exposed mice showed trends toward altered *Ltbr* promoter DNA methylation in the cerebellum. Fold change in expression over *Pgk1* and percent methylation of *Ltbr* promoter for: **(A)** female cortex; **(B)** male cortex; **(C)** female cerebellum; **(D)** male cerebellum. Blue: male; orange: female. All expression and DNA methylation comparisons were nonsignificant (Student's t test). Error bars: SEM.

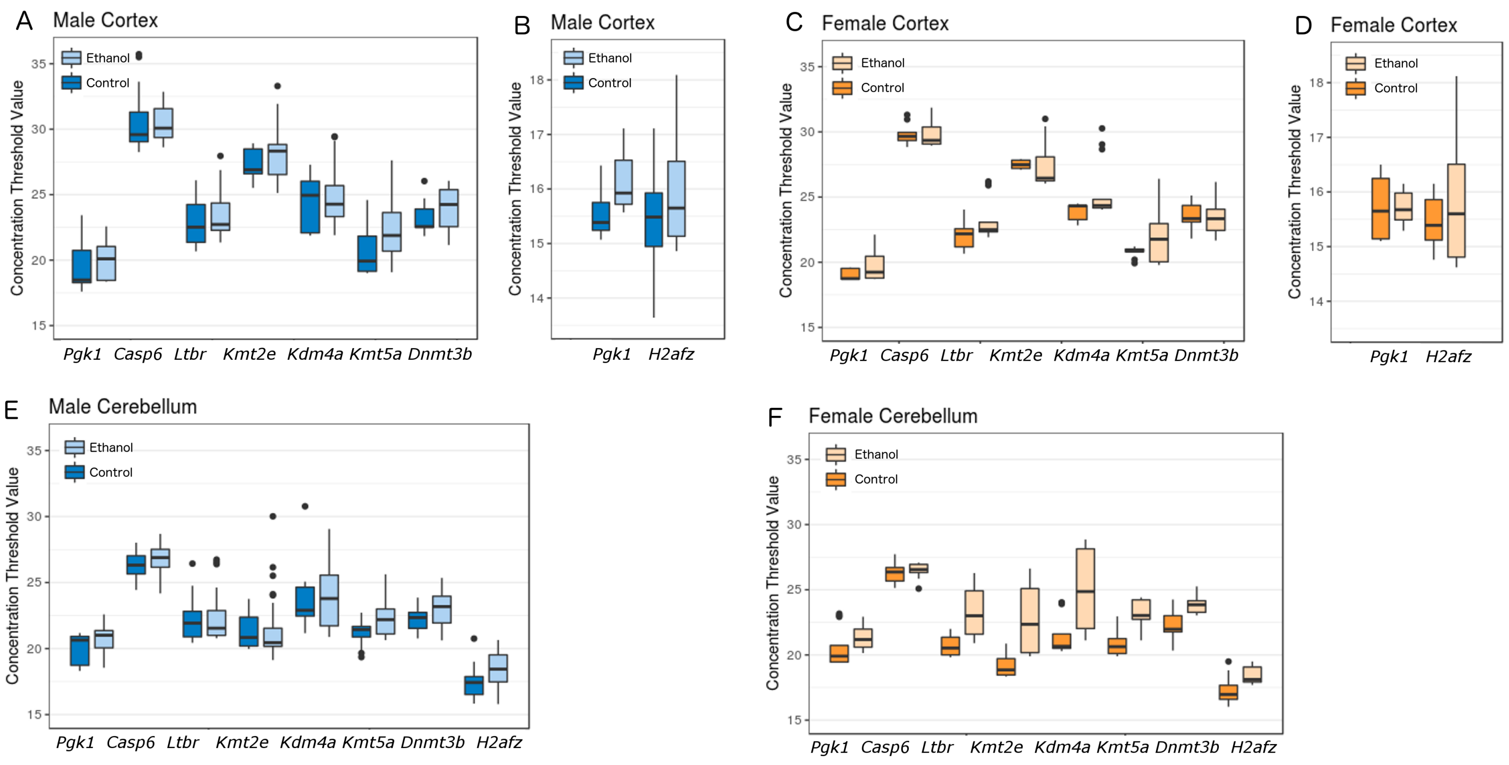

Supplementary Figure 4. Concentration threshold values prior to *Pgk1* normalization. Average concentration threshold (CT) values of the final 7-gene panel plus *Pgk1* for: **(A)** male cortex, 1:100 dilutions; **(B)** male cortex, 1:10 dilutions; **(C)** female cortex, 1:100 dilutions; **(D)** female cortex, 1:10 dilutions; **(E)** male cerebellum; **(F)** female cerebellum. Blue: male; orange: female. Higher CT value indicates lower absolute expression.

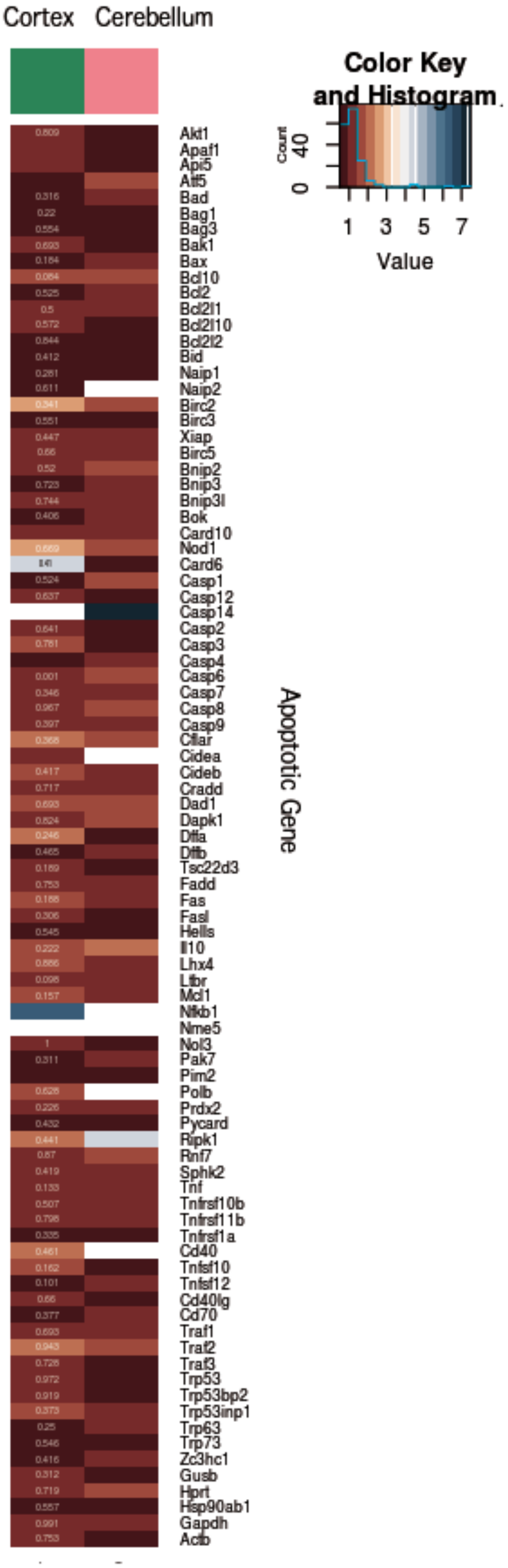

Supplementary Figure 5. Ethanol expression fold change values for qPCR array apoptotic genes. Heat map displaying expression fold change (cell fill colour) for ethanol-treated mice over control across 84 apoptotic genes on the SABiosciences Mouse "Apoptosis" qPCR array (cortex n=3/group, cerebellum n=2/group, all male). Data was normalized to the geometric mean of five genes (*Gusb*, *Hprt*, *Hsp90ab1*, *Gapdh*, and *Actb*). Cell text: Student's t-test p-value; green: cortex; pink: cerebellum.
